# NLRP3 Activation and Its Relationship to Endothelial Dysfunction and Oxidative Stress: Implications for Preeclampsia and Pharmacological Interventions

**DOI:** 10.3390/cells10112828

**Published:** 2021-10-21

**Authors:** Priscila Rezeck Nunes, Sarah Viana Mattioli, Valeria Cristina Sandrim

**Affiliations:** Department of Biophysics and Pharmacology, Institute of Biosciences, Sao Paulo State University, Sao Paulo 01049-010, Brazil; sarah.mattioli@unesp.br (S.V.M.); valeria.sandrim@unesp.br (V.C.S.)

**Keywords:** NLRP3, preeclampsia, nitric oxide, endothelial dysfunction, oxidative stress, inflammation

## Abstract

Preeclampsia (PE) is a specific syndrome of human pregnancy, being one of the main causes of maternal death. Persistent inflammation in the endothelium stimulates the secretion of several inflammatory mediators, activating different signaling patterns. One of these mechanisms is related to NLRP3 activation, initiated by high levels of danger signals such as cholesterol, urate, and glucose, producing IL-1, IL-18, and cell death by pyroptosis. Furthermore, reactive oxygen species (ROS), act as an intermediate to activate NLRP3, contributing to subsequent inflammatory cascades and cell damage. Moreover, increased production of ROS may elevate nitric oxide (NO) catabolism and consequently decrease NO bioavailability. NO has many roles in immune responses, including the regulation of signaling cascades. At the site of inflammation, vascular endothelium is crucial in the regulation of systemic inflammation with important implications for homeostasis. In this review, we present the important role of NLRP3 activation in exacerbating oxidative stress and endothelial dysfunction. Considering that the causes related to these processes and inflammation in PE remain a challenge for clinical practice, the use of drugs related to inhibition of the NLRP3 may be a good option for future solutions for this disease.

## 1. Introduction

Preeclampsia (PE) is a specific syndrome of human pregnancy, considered the main cause of morbidity and mortality in 2 to 8% of pregnancies worldwide [1], and one of the main causes of maternal death. The clinical parameters that characterize this pathology are arterial hypertension and proteinuria from the twentieth week of pregnancy or in the first days after delivery. However, other maternal dysfunctions may also be related to PE, such as renal failure, liver involvement, neurological or hematological complications, uteroplacental dysfunction, or fetal growth restriction [2,3]. This pathology increases the risk of maternal and fetal mortality, through placental abruption, cerebrovascular events, organ failure, and disseminated intravascular coagulation [4].

In a healthy pregnancy, during the second trimester of gestation, maternal spiral arteries are invaded by the trophoblast that phenotypically differentiates into endothelial cells, resulting in remodeling of the spiral arteries [5]. Vasculogenesis ensures adequate blood supply to the placenta and fetal growth, however, it is observed that in placentas of pregnant women with PE, trophoblastic invasion is inadequate, occurring in only 30–50% of the arteries [6]. This failure in vascular remodeling can lead to poor placental perfusion and ischemia [7]. Ischemia occurs since the arteries are not sufficiently remodeled, causing disorderly perfusion of blood flow to the intervillous space. Added to an inadequate supply of nutrients and oxygen, there is a reduction in the surface area available for exchange between mother and fetus, which can contribute to unfavorable pregnancy-related outcomes [8]. The pathophysiology of PE is not fully understood, but it is currently known that placental ischemia is of fundamental importance in this process, since the release of products resulting from poor perfusion in the maternal circulation can lead to systemic endothelial dysfunction [9].

Endothelial cells have different functions during non-inflammatory conditions, such as maintaining blood fluidity, regulating blood flow, and maintaining leukocytes in a basal state circulating [10]. In cases of infection or inflammation, these cells recognize danger signals and they act as active regulators of the inflammatory response [10], and receptors in these cells help the response to a range of external signals [11]. This meeting between endothelial cells and danger signals, such as ATP and high mobility group box 1 protein (HMGB1), can activate the NOD-like receptor family, pyrin domain-containing protein 3 (NLRP3) [12,13]. Activation of NLRP3 inflammasome in endothelial cells was already observed in animal models, and production of IL-1β by these cells has been shown to contribute to diverse pathological conditions [14,15].

Several recent studies in the literature have demonstrated that women with PE present a significantly higher expression of NLRP3, and related mediators such as caspase-1, IL-1, and IL-18 compared to normotensive healthy pregnant women [16,17,18].

This article aims to summarize the role of NLRP3 in PE related to endothelial dysfunction and oxidative stress, proposing different approaches for future therapies.

## 2. Preeclampsia and Endothelial Dysfunction

Endothelial cells form a monolayer that covers the interior of blood vessels, creating a barrier between blood and the extravascular matrix. These cells have a fundamental role in maintaining a dynamic modulation of homeostasis, angiogenesis, and vascular tone, besides maintaining an antioxidant, anti-inflammatory, and antithrombotic profile in healthy individuals [19]. Endothelial dysfunction is the term used to describe an imbalance in these endothelial functions affecting vasoprotective homeostasis [20].

In normal pregnancies, the typical increase in blood volume is commonly compensated by a slight decrease in blood pressure. For a long time, this rise in blood pressure has been associated with reduced maternal vascular resistance [21,22]. However, in PE, the compensatory maternal vascular adaptations are insufficient, and it has been associated with systemic endothelial dysfunction [23,24,25,26]. In this syndrome, this characteristic is associated with the PE poorly perfused placenta, which releases proinflammatory and antiangiogenetic factors into maternal circulation [27,28]. This hypothesis has been reinforced by many studies so far. For example, Myers and colleagues demonstrated that healthy myometrium vessels incubated with plasma from PE pregnant women had reduced endothelium mediated vascular relaxation compared to those incubated with plasma from healthy pregnant women [29]. Plasma from women with PE can modify the endothelial function, altering the balance between vasoactive substances. Despite evidence showing the release of placental factors into maternal circulation could alter endothelial function, the exact mechanism is not fully understood. [30].

Until today, many studies demonstrated several alterations, both locally and in circulation, in multiple bioactive factors in PE. For example, the angiogenic balance disturbance has been described by decreases in pro-angiogenic vascular endothelial growth factor (VEGF) and placental growth factor (PlGF) by the action of the placental soluble fms-like tyrosine kinase-1 (sFlt-1) and soluble endoglin (sEng) [29,31,32,33]. Moreover, proinflammatory molecules such as tumor necrosis factor-α (TNF-α), endocan, interleukin-6 (IL-6), and IL-1β have also been reported to be altered in PE [34,35,36,37]. Altogether, these alterations lead to systemic endothelial dysfunction in PE, and it also is possible that there may be more mechanisms involved that were not discovered yet.

These alterations seem to contribute to the development of the significant symptoms of the maternal syndrome in PE, such as hypertension, edema, proteinuria, and platelet aggregation [38]. For example, the decreased levels of NO production and increased levels of ET-1 and sFlt-1 leads towards a vasoconstrictive and hypertensive maternal profile [39]. Regarding endothelial monolayer barrier integrity, the reduced levels of pro-angiogenic and increased levels of proinflammatory molecules lead to a more permeable profile of the vascular endothelium, which may lead to edema, proteinuria, and even cerebral endotheliosis, that leads to seizures in severe cases [40].

The different mechanisms overlapping each other lead to a common end, endothelial dysfunction, and this condition represent a major hallmark of PE, contributing to the clinical consequences of the disease. Therefore, fully understanding and identifying the factors that lead to endothelial damage is the key to further understand the pathogenesis of PE and provide early diagnosis and effective therapies.

## 3. NLRP3 Inflammasome Activation and Regulation in Preeclampsia

### 3.1. Inflammasome Formation and the Role of NLRP3 in the Pathogenesis of PE

The immune response is divided into innate and adaptive immunity. Contact with pathogens or any danger signal activates the innate immune system, as the first line of defense. This process starts quickly as possible to protect the organism and to maintain homeostasis. The immune cells detect the signals from invaders, expressing molecules known as pathogen-associated molecular patterns (PAMPs). Besides that, these cells also identify molecules associated with inflammation and cell death, in cases of sterile inflammation, without any external microbial sign. These molecules associated with inflammation are named damage-associated molecular patterns (DAMPs). PAMPs and DAMPs are recognized by pattern recognition receptors (PRRs). Two of the most studied PRRs are Toll-like receptors (TLRs) and nucleotide-binding domain leucine-rich repeat-containing receptors (NLRs) [41].

There are 22 recognized members of the NLR family, between them, NLRP3 (NOD-like receptor family, pyrin domain-containing protein 3) is the most studied and investigated, because this NLR forms complexes with other proteins, forming multimeric complexes, called inflammasomes [42].

**Figure 1:**
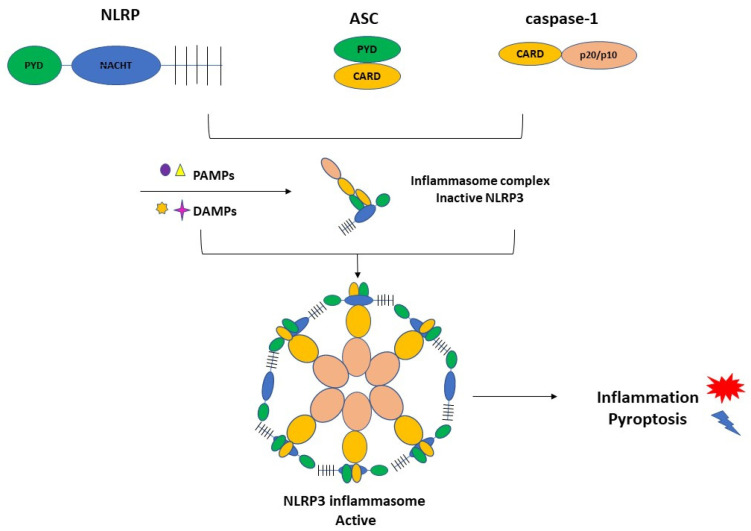
The NLRP3 inflammasome consists of NLRP3, ASC, and caspase-1. NLRP3 is composed of C-terminal leucine-rich repeats (LRRs), a central nucleotide-binding and oligomerization domain (NACHT), and an N-terminal pyrin domain (PYD). ASC is also termed Pycard, containing an N-terminal PYD and a C-terminal caspase recruitment domain (CARD). The last element of the CARD and caspase domains. PAMPs and DAMPs can activate the inflammasome complex and triggers inflammation and pyroptosis.

NLRP3 inflammasome complex is constituted by NLRP3, ASC (apoptosis-associated speck-like protein containing a caspase recruitment domain), and the cysteine protease precursor procaspase-1 (Figure 1).

Recently, reports regarding the NLRP3 inflammasome activation in PE have been increased. The literature shows higher expression of the NLRP3 inflammasome components in blood cells and placenta from PE women compared with normotensive healthy pregnant women [16,43,44]. Furthermore, trophoblastic cells also express NLRP3, ASC, and caspase-1 [45,46,47], and IL-1β secretion occurs in human trophoblast cells in response to activators of the NLRP3 inflammasome [46,47]. The interaction between alarmin-induced activation of placental NLRP3 inflammasome and the resulting placental inflammation presented in pregnancy complications such as preeclampsia has been shown by in vivo studies [46,48,49,50].

These recent contributions suggest that NLRP3 inflammasome activation is implicated in the inflammatory processes associated with the pathophysiology of preeclampsia. Moreover, in vitro and in vivo studies have shown that inflammatory stimuli induce the activation of the NLRP3 inflammasome in the placenta, also contributing to other pregnancy-related disorders [51].

### 3.2. Activation of NLRP3 and Pyroptosis: The Cell Death Related to Inflammatory Processes

The literature data showed significantly higher expression of the NLRP3 and related mediators such as caspase-1, IL-1, and IL-18 in samples from women with PE compared to controls [16,44]. Other groups highlighted the NLRP3 gene polymorphisms associated with a significantly higher risk of disease development [17,18].

**Figure 2:**
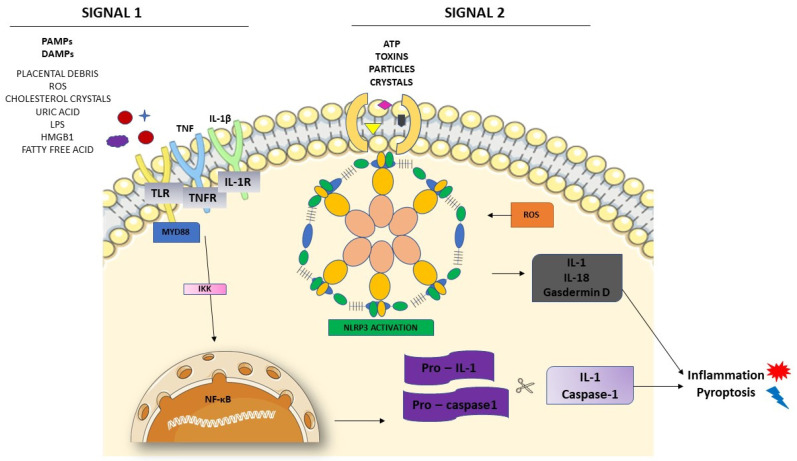
NLRP3 inflammasome activation. The priming signal (signal 1) occurs in the presence of danger signals (PAMPs and DAMPs), leading to the activation of the NF-κB and subsequent upregulation of NLRP3 and pro-IL-1 and pro-caspase1. The activation signal (signal 2) starts with the direct activation of the NLRP3 inflammasome with ROS recruitment. The process leads to inflammation and pyroptosis.

Inflammasome activation starts with two signals, both initiated by DAMPs or PAMPs [52,53]. Figure 2 shows these two different signals in the activation of NLRP3. The first one is the priming signal, leading nuclear factor kappa B (NF-κB) activation through membrane receptors. NF-κB is important in the activation of the transcription and regulators of several genes, inducing the expression of pro-IL-1 and NLRP3. In the second signal, PAMPs and DAMPs appear to bind directly to NLRP3 [53]. Once activated, NLRP3 interacts with ASC, recruiting and activating procaspase-1. The interaction between NLRP3 and ASC activates caspase-1, as well as pro-IL-1 and IL-18, releasing these cytokines in their active forms. 

Pyroptosis, a programmed necrosis type, involves recruitment of its executor gasdermin D, (GSDMD) leading to inflammatory cascades, releasing alarmins or DAMPs. Besides cleavage of pro-IL-18/pro-IL-1β in inflammasome activation, Cheng et al., 2019 also demonstrated that pro-GSDMD is also cleaved into N-terminal which are translocated to the plasma membrane and form pores, which leads to pyroptosis and subsequent release of cell particulates, including DAMPs. GSDMD is significantly expressed in the placenta from early-onset PE and in cellular models of PE pathophysiology. They concluded that placental pyroptosis is a major sterile inflammatory pathway in PE that may lead to excessive production of IL-1β and IL-18, contributing to the systemic manifestation of this disease [54].

This type of cell death-related with NLRP3 activation is caspase-1-dependent because it depends on plasma membrane rupture. This process releases DAMPs and cytokines into the extracellular milieu, leading to sterile inflammation, as it occurs in PE. This type of programmed cell death generates highly inflammatory species [55]. This process releases IL-1β, IL-18, and HMGB1 (high mobility group box 1), which distinguishes this type of cell death from others. Pyroptosis has been identified as a potent cause of endothelial cell death [56].

## 4. NLRP3 and its Relation with Endothelial Dysfunction and Oxidative Stress

According to Burton et al., 2019, the release of products resulting from poor perfusion in the maternal circulation can lead to systemic endothelial dysfunction [9]. The mechanism by which these products are released into the maternal circulation, how they modify endothelial function in pregnant women with PE, how they change the balance between vasoactive substances, such as NO, prostacyclin, and endothelin, is not yet fully understood [30]. The literature data suggest that the generalized endothelial dysfunction seen in PE is the main cause of the clinical abnormalities observed in this disease [38,57]. Vascular endothelial cells cover the inner layer of blood vessels, forming a barrier between blood and the extravascular matrix. This barrier maintains the transport of solutes, fluids, and cells [58]. Endothelial barrier dysfunction is characterized by loss of contact between endothelial cells and the extravasation of plasma, proteins, cells, and solutes [59].

The products resulting from endothelial dysfunction can act as inflammatory mediators, activating the innate immune system, the first mechanism by which the body responds immediately to infections and injuries [60]. Cells from the innate immune system play an important role in the inflammatory response initiated by PRRs, but cells outside the immune system, such as endothelial cells, still need to be better studied in this process [61].

Generally, DAMPs can trigger NLRP3 inflammasome activation, producing mature forms of IL-1β and IL-18 from cells to promote further inflammatory processes and oxidative stress in the endothelium [62]. Endothelial cells (ECs) are a target of IL-1β, and it also produces IL-1β during inflammation [63], activating other inflammatory mediators, contributing to secreting adhesion molecules and chemokines in ECs, inducing a potent pro-inflammatory response [64]. Endothelial inflammation may initiate the occurrence and progression of endothelial dysfunction.

**Figure 3:**
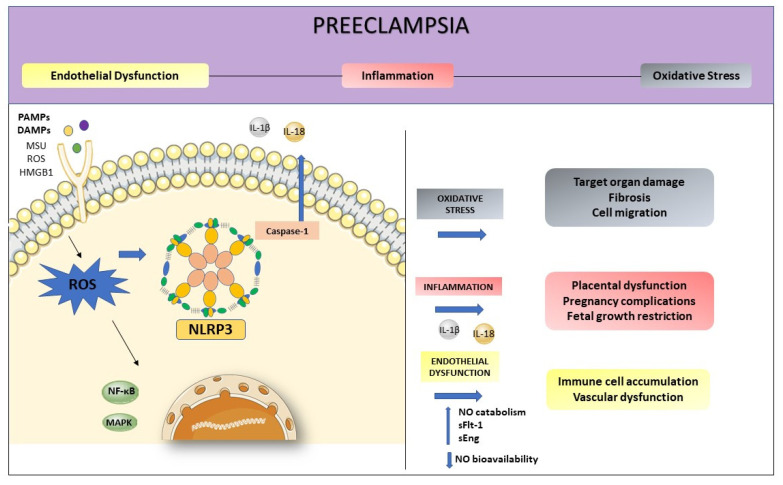
Preeclampsia is characterized by intense oxidative stress, inflammation, and endothelial dysfunction. The activation of NLRP3 may start with the production of ROS. Inflammasome activation is responsible for the release of inflammatory agents during the immune response, such as IL-1β and IL-18. High levels of ROS increase NO catabolism and decrease NO bioavailability as well as increasing factors such as sFlt-1 and sEng. This process enhanced inflammation-related genes expression, contributing to endothelial dysfunction.

Oxidative stress and inflammation are inseparable events in inflammatory diseases and both play an essential role in the pathogenesis of PE (Figure 3). The NLRP3 activation initiates from various stimuli, including the production of reactive oxygen species (ROS) [65]. They are the first intermediate reactive products generated during inflammasome activation, being responsible for the release of inflammatory agents in the immune response [66]. 

In this way, ROS mediate the interaction between NLRP3 inflammasome and endothelial dysfunction, being the first participant in the NLRP3 activation, promoting inflammation, and activating immune responses [66]. Three important proteins, thioredoxin-interacting protein (TXNIP), nuclear factor kappaB (NF-κB), and the transcription factor nuclear factor erythroid 2-related factor 2 (Nrf2) are involved in the oxidative stress, connecting ROS to NLRP3 activation [67]. In a state of increased oxidative stress, as occurs in preeclampsia, the imbalance between pro and antioxidants, coupled with higher ROS production may increase NO catabolism, and decrease NO bioavailability. The oxidative stress enhanced inflammation-related genes expression and increased inflammatory proteins, impairing endothelial function [68].

## 5. Pharmacological Interventions: Selective and Non-Selective Drugs

The association of NLRP3 inflammasome activation with various inflammatory diseases involves interest in the scientific community to explore the actions of the effective NLRP3 inflammasome inhibitors. Several inhibitors of NLRP3 inflammasome have been reported. Here, we summarize recent pharmacological inhibitors in Table 1. A diverse range of targets can be used for its inhibition due to the fact of its complex signaling cascade.

Different strategies may be used for inflammasome inhibition, such as suppression of activation signals, blockade of inflammasome complex formation, inhibition of caspase-1 activation, blockade of pore-forming protein gasdermin D, avoid inflammatory cytokines production and release. Here, we describe some drugs that are summarized in Table 1.

Glyburide, also known as glibenclamide, is a drug from the sulfonylurea family widely prescribed to treat type 2 diabetes mellitus (T2D), and it is suggested to effectively inhibit the migration of inflammatory cells, as it prevents the assembly of the inflammasome. Specifically, glibenclamide inhibits NLRP3 activation by inducing the closure of ATP-sensitive potassium channels, increasing the intracellular potassium concentration [69].

Thus, there is a reduction in inflammatory cell infiltration, preventing further organ damage in ischemic tissue [70,71]. This drug works by improving endothelial dysfunction and has also been described as an inhibitor of NLRP3 in endothelial cells in the blood–brain barrier [17]. Furthermore, some authors have shown that this drug was able to cause vessel relaxation in vascular reactivity studies in rats [72]. Studies with glyburide administration in vitro or in vivo [69,73,74,75] showed inhibitory activity of NLRP3 activation. However, the necessary dose in vivo is high to exert an inhibitory effect and can cause hypoglycemia. Because of that, the use of glyburide is still restricted to T2D [75]. 16673-34-0 is a glyburide intermediate substrate produced during its synthesis and appears not to affect glucose metabolism. A study conducted by Marchetti et al. showed that 16673-34-0 inhibits NLRP3 inflammasome formation in murine macrophages and rat cardiomyocytes. In vivo tests showed positive results in mouse models of acute myocardial infarction. This substrate was tested in the presence of diverse stimuli of the NLRP3 inflammasome, and independent of the stimuli, the inhibitory effects of 16673-34-0 remained the same. This information suggests that this molecule interferes with downstream events involved in both NLRP3 activation and binding to ASC [71,72].

A novel small molecule developed by Kuwar et al., named JC124, mimetics the structure of glyburide and attends to minimize the hypoglycemic effects of glyburide. It was tested in traumatic brain injury (TBI) therapy and exerted a significant anti-inflammatory effect to protect the injured brain. Treatment with this molecule reduced the expression of NLRP3, ASC, caspase-1, pro-IL-1β, TNFα, and inducible nitric oxide synthase (iNOS) [76]. Besides that, JC124 also showed protective effects in a mouse model of acute myocardial infarction [77].

Another synthetic molecule created by Liu et al., 1-ethyl-5-methyl-2-phenyl-1H-benzodimidazole, also known as FC11A-2, has inhibitory potential for NLRP3 inflammasome. This molecule was tested in THP-1 cells and a mouse model of experimental colitis, showing a blockage in IL-1β/18 release and reduced activated caspase-1, in an NF-κB independent pathway [78].

A selective NLRP3 inhibitor, MCC950, specifically acts to inhibit this inflammasome. This drug blocks the oligomerization of ASC and the hydrolysis of ATP [79,80] and has been studied in several human diseases, proving to be effective in the treatment of vascular dysfunction in diabetes [81], and sepsis [82,83]. This inhibitor was reported to decreased inflammation in skin and lungs in mice [84] and some other in vivo experiments showed that MCC950 alleviates the severity of experimental autoimmune encephalomyelitis (EAE) [79].

CY-09 is described as an analog of CFTR (inh)-172 (C172), inhibiting the cystic fibrosis transmembrane conductance regulator (CFTR) channel [85]. Jiang et al. identified an effect of this molecule in NLRP3 activation with significant inhibition in vivo in mice models and ex vivo in human cells [86]. CY-09 acts blocking the ATP, MSU, and nigericin-induced activation of caspase-1 and consequently IL-1β release. CY-09 demonstrated preventive and therapeutic actions in the mice models of gout, T2D, and cryopyrin-associated periodic syndromes (CAPS). A great achievement about this molecule is that CY-09 showed good oral bioavailability, safety, and stability [87].

Tranilast (N-[3′,4′-dimethoxycinnamoyl]-anthranilic acid, also known as TR) is a tryptophan metabolite analog [88]. TR prevents the interaction between NLRP3 and ASC, proving that it affects the NLRP3 activation directly. TR has already been demonstrated as a significant therapy for the prevention of poor outcomes in gout, CAPS, and T2D mice models [80]. This drug has an important aspect that is the safety in high doses with appropriate tolerance levels in patients [89,90] This tolerance is important because it allows the use for tests in NLRP3-related diseases treatments.

OLT1177 is an active β-sulfonyl nitrile compound used in experimental clinical tests for the treatment of degenerative arthritis [91]. OLT1177 blocked in vitro activation of NLRP3 and direct binding with NLRP3 to block its ATPase activity. This drug was already given orally in clinical trials, showing safety and tolerability. OLT1177 presents a long half-life and did not show organ or hematological toxicity [92]. Thus, it seems to show significant potential for the treatment of NLRP3-related disorders.

Oridonin is a bioactive compound of Rabdosia rubescens, which is extensively utilized in traditional Chinese medicine [93,94]. This drug acts by inhibiting the NF-κB/MAPK activation and the release of inflammasome-independent proinflammatory cytokines [95,96]. This drug was tested in mice models of T2D, peritonitis, and gouty arthritis, exhibiting significant preventive, and therapeutic effects [97]. It could be used in future studies as a clinically applicable inhibitor of NLRP3 inflammasome.

A plant sesquiterpene lactone named Parthenolide has numerous anti-inflammatory effects and has been utilized in herbal medicine for the treatment of various inflammatory diseases [98]. It acts by inhibiting caspase-1 activation in response to NLRP1, NLRC4, and NLRP3 activation via caspase-1. This drug can also target the ATPase activity of NLRP3 [99]. The main concern for the use of Parthenolide is the poor solubility and bioavailability [100,101].

VX-740 (also known as Pralnacasan) and its analog VX-765 are inhibitors of caspase-1 [102,103], blocking this protein and resultant cleavage of pro-IL-1β/18 [104]. In rheumatoid arthritis (RA) clinical trials, these pro-drugs exhibited significant anti-inflammatory effects with a good pharmacokinetic profile [105,106]. Moreover, it had positive outcomes for the treatment of epilepsy and psoriasis in mouse models nonetheless, hepatic toxicity in animals after long-term exposure remains a concern [103,107,108].

Bay 11-7082 is a phenyl vinyl sulfone and acts inhibiting the NF-κB pathway [87]. Tests with NG5 cells and mouse primary bone marrow-derived macrophages (BMDMs) showed that this drug prevents the organization of ASC pyroptosome and NLRP3 inflammasome. Initial clinical trials showed that these compounds are well-tolerated, non-mutagenic, with suitable pharmacokinetic profiles, as well as also having the ability to permeate the cell membrane easily [99].

The last drug listed here is β-hydroxybutyrate (BHB), a ketone metabolite, which was tested for NLRP3 inflammasome blockade by Youm et al. BHB was able to decrease the production of IL-1ß and IL-18 in human monocytes in response to activated NLRP3 inflammasome. It blocks the activation of NLRP3 inflammasome independent of ROS, AMP-activated protein kinase, glycolytic inhibition, or autophagy [109]. Thus, BHB could be used in trials to reduce the severity of NLRP3-mediated chronic inflammatory diseases.

Many inhibitors for NLRP3 inflammasome have been reported in the literature and some of them have shown remarkable therapeutic potential. More research is needed to develop specific and safe molecules to inhibit NLRP3 inflammasome. The use of drugs with clinical positive results may be the ideal choice for the treatment of endothelial dysfunction, providing a new strategy to treat related illnesses. Considering that the causes related to endothelial dysfunction, oxidative stress, and inflammation in PE remain a challenge for clinical practice, the use of pharmacological substances related to the inhibition of the NLRP3 inflammasome may be a good choice to propose future treatments and strategies for PE. In addition, expanding research into the role of NLRP3 in endothelial dysfunction may enrich the understanding of several inflammatory diseases.

## 6. Conclusions

NLRP3 activation plays an important role in the development of PE. Although NLRP3 has been the most intensively investigated type of inflammasome, a total mechanism for its activation has not yet been elucidated. Therefore, inhibitors of NLRP3 could be a very effective treatment for PE. With new research, the mechanisms regarding endothelial function and its relation to the NLRP3 inflammasome activation pathway can be better elucidated. Meanwhile, the interactions between endothelial dysfunction, oxidative stress, and the NLRP3 inflammasome-regulated pathways may improve the treatments of inflammation-related disorders, such as PE.

## Figures and Tables

**Table 1:** Potential inhibitors of NLRP3 inflammasome. NLRP3-specificity and targets (including the mode of action) are also represented.

Drug	NLRP3-Specific	Direct Inhibition	Action
Glyburide [17,69,70,71,72,73,74,75]	Yes	No	Induces the closure of ATP-sensitive K+ channels; Raises the intracellular K+ concentration
16673-34-0 [71,72]	Yes	No	Interferes with downstream events involved in NLRP3 conformational changes secondary to activation or binding to ASC
JC124 [76,77]	Yes	No	Blocks ASC aggregation, caspase-1 activation, and IL-1β secretion
FC11A-2 [78]	Yes	No	Repress IL-1β/18 release; induces autocleavage of procaspase-1, resulting in a reduced amount of activated caspase-1
MCC950 [79,80,81,82,83,84]	Yes	Yes	Blocks the release of IL-1β induced by NLRP3 activators
CY-09 [85,86,87]	Yes	Yes	Blocks the ATP, monosodium urate (MSU), and nigericin-induced activation of caspase-1 and resultant release of IL-1β
Tranilast [80,88,89,90]	Yes	Yes	Impairs the endogenous NLRP3-ASC interaction
OLT1177 [91,92]	Yes	Yes	Binds with NLRP3 to block its ATPase activity
Oridonin [93,94,95,96,97]	Yes	Yes	Inhibits the NF-κB or MAPK activation and repress the release of inflammasome-independent proinflammatory cytokines release
Parthenolide [98,99,100,101]	No	No	Inhibits caspase-1 activation; Targets ATPase activity of NLRP3
VX-740/VX-765 [102,103,104,105,106,107,108]	No	No	Block caspase-1 and resultant cleavage of pro-IL-1β/18
Bay 11-7082 [87,99]	No	No	Prevents the organization of ASC pyroptosome
BHB [109]	No	No	Lowered the production of IL-1ß and IL-1; reduces the oligomerization and speck formation of ASC

## Data Availability

Not applicable.
